# Risk factors for the recurrence of instability after operative treatment of chronic lateral ankle instability: A systematic review

**DOI:** 10.1002/jeo2.70214

**Published:** 2025-03-22

**Authors:** Ronny Lopes, Choon Chiet Hong, James Calder, Gino M. M. J. Kerkhoffs

**Affiliations:** ^1^ Department of Orthopaedic Surgery and Sports Medicine Centre Orthopédique Santy, FIFA Medical Centre of Excellence, Groupe Ramsay‐ Generale de Sante, Hôpital Privé Jean Mermoz Lyon France; ^2^ Department of Orthopaedic Surgery National University Hospital Singapore; ^3^ Department of Orthopaedic Surgery, Yong Loo Lin School of Medicine National University of Singapore Singapore; ^4^ Fortius Clinic (FIFA Medical Centre of Excellence) London UK; ^5^ Department of Bioengineering Imperial College London London UK; ^6^ Department of Orthopedic Surgery and Sports Medicine Amsterdam Movement Sciences, Amsterdam University Medical Centers Amsterdam The Netherlands

**Keywords:** ankle instability, lateral ankle ligament repair, recurrence of instability, risk factors, systematic review, treatment failure

## Abstract

**Purpose:**

To identify, review and summarize risk factors for failure of lateral ankle ligament operative treatment for chronic lateral ankle instability (CLAI).

**Methods:**

A Systematic review according to PRISMA guidelines was performed. In July 2023, a bibliographic search of the PubMed, Medline, CINAHL, Cochrane, and Embase databases was performed. Articles were included if they were quantitative studies published in English and reported risk factors for recurrence of instability.

**Results:**

A total of 496 articles were identified using the search strategy, and nine articles were included. All were low‐quality cohort studies (level 3 or 4 evidence). These nine studies comprising 762 participants met the criteria for inclusion. Eighty‐nine patients (11%) had treatment failure as defined by recurrence of instability, with rates ranging from 5.7% to 28.5%. Six risk factors were divided into three categories: patient demographics (generalized joint laxity [GJL], high‐level sports activities and female sex), imaging features (varus hindfoot alignment), and surgical findings (poor quality of the remnant lateral ligaments, intraoperative syndesmosis widening).

**Conclusion:**

The presence of risk factors such as GJL, high‐level sports activities, female sex, varus hindfoot alignment, poor ligament quality, and intraoperative syndesmosis widening should guide surgical strategy to reduce the risk of treatment failure in lateral ankle ligament repair for CLAI.

**Level of Evidence:**

Level IV, systematic review.

**Abbreviations**
AAS: ankle activity score
ATFL: anterior talofibular ligament
BMI: body mass index
CFL: calcaneofibular ligament
CINAHL: Cumulative Index to Nursing & Allied Health Literature
CLAI: chronic lateral ankle instability
FAAM: Foot and Ankle Ability Measure
FAOS: foot and ankle outcome score
GJL: generalized joint laxity
MeSH: Medical Subject Headings
PRISMA: Preferred Reporting Items for Systematic reviews and Meta‐Analyses
SAFE‐Q: Self‐Administered Foot Evaluation Questionnaire

## INTRODUCTION

Lateral ankle sprain is amongst the most common musculoskeletal injuries. Its incidence is estimated between 2.1 and 3.2 per 1000 person‐years [21] and it is more prevalent in the active sporting population [22]. Despite medical treatment and prevention [26, 51], lateral ankle sprain recurrence rates in sports such as basketball have been reported to exceed 70%. These injuries may lead to residual disabilities, including pain, swelling, recurrent sprains, and chronic lateral ankle instability (CLAI) [22]. CLAI is one of the most common complications, occurring in 40% of cases [13, 20].

Various terms have been used to describe this condition, such as CLAI, functional ankle instability, mechanical ankle instability, and recurrent ankle instability. Among these, chronic ankle instability is most commonly defined as a broad term that includes both mechanical and functional instability of the ankle joint.

For individuals with CLAI who fail conservative management with functional rehabilitation surgical treatment may be considered as this has been shown to produce positive long‐term outcomes [5, 6]. Although numerous surgical techniques have been described [49], surgical stabilization of CLAI may be broadly divided into lateral ligament repair or ligament reconstruction [36]. As the name suggests, ligament repair involves repairing the lateral ligament(s) via anatomical direct or indirect repair, whereas ligament reconstruction uses a tendon graft (autograft/allograft) to reconstruct one or two of the lateral ligaments [2].

The modified Broström–Gould [18] procedure is a direct ligament repair technique considered to be the first‐line treatment in the surgical management of CLAI and often regarded as the gold standard treatment [11]. However, treatment failures have been described in some series, ranging from 0% to 32% of cases [4, 5, 6, 8, 25, 32, 34]. One reason for this wide variation in failure rate may be explained by the difficulty of defining ankle instability [15]. A recent systematic review has reviewed 42 studies and found a plethora of definitions in the literature to describe surgical failures after surgery for lateral ankle instability [12].

Similarly, the literature is lacking in the risk factors for failure of lateral ankle ligament repairs. Understanding the cause for failure is difficult because accurate information is lacking and contradictory results are reported regarding specific risk factors for treatment failure [41, 55]. Furthermore, although some of these risk factors are generally accepted in clinical practice such as generalized joint laxity and hindfoot varus [19], they do not necessarily guide the surgical decision [38].

Recently, several studies [33, 46, 48, 55, 56] report on the risk factors for failure of lateral ankle ligament repairs but to date, no study has reviewed the literature on these risk factors. Therefore, the aim of this study is to identify, review and summarize these commonly cited risk factors for failure of lateral ligament repair techniques in CLAI. We hypothesize that reviewing and summarizing these risk factors will offer valuable insights to improve surgical decision‐making and enhance patient outcomes in cases of CLAI.

## MATERIALS AND METHODS

### Search strategy

The structure of this review followed the recommendations on systematic reviews of literature and meta‐analyses [24, 39]. The objectives, analytic methods and inclusion criteria were determined before collecting data by following the Preferred Reporting Items for Systematic reviews and Meta‐Analyses (PRISMA) recommendations. In July 2023, a bibliographic search of the PubMed, Medline, Cumulative Index to Nursing and Allied Health Literature (CINAHL), Cochrane, and Embase databases was done. The search strategy used for PubMed, which was modified for other database, was as follows: using the Medical Subject Headings (MeSH) terms: [(ankle repair OR Broström OR ankle reconstruction OR ankle ligament repair OR ankle ligament Broström OR ankle ligament reconstruction OR lateral ankle ligament repair OR lateral ankle ligament Broström OR lateral ankle ligament reconstruction OR anterior talofibular ligament (ATFL) repair OR ATFL Broström OR ATFL reconstruction OR ATFL calcaneofibular ligament (CFL) repair OR ATFL CFL Broström OR ATFL CFL reconstruction) AND (failure OR recurrence OR reinjury)] AND (English[Language]).The initial selection of articles based on the title and abstract was carried out by two of the authors (R.L. and C.C.H.) independently. If there was disagreement about the status of an article, authors discussed it to come to a consensus. A second filtering step was applied by reading the entire article and reviewing the reference list of each selected article to make sure that no article on this topic had been overlooked. The selected studies were written in English only, with no time restriction on the publication date up to July 2023 and had an abstract available online.

### Inclusion and exclusion criteria

The studies included met the following criteria:

‐ Reported the risk factors for the failure of treatment after lateral ankle ligament repair defined as recurrence of instability (recurrence of instability was taken as the definition in this study because it was the most commonly cited definition of treatment failure in the literature [18] as well as it is in the authors' belief that the recurrence of instability would simply mean the failure of surgical repair to meet its primary objective)
‐ Provided follow‐up results of at least 1 year.
‐ Written in English language.

The following were the exclusion criteria:

‐ Type of articles (review articles, meta‐analysis, case reports, editorial, technique articles, biomechanical studies, cadaveric studies or animal experiments).
‐ Insufficient follow‐up duration.
‐ Abstract from a meeting/conference.
‐ Duplicated studies.

### Data extraction and analysis

Two reviewers independently extracted data from each study. After selecting the papers for inclusion in the systematic review, the following data were extracted: level of evidence [52], total number of patients, patient's demographic data, follow‐up duration, surgical technique, recurrent instability rate, and risk factors for failure. Additionally, the risk factors were categorized into three groups (patient factors, imaging assessments, and surgical findings) to facilitate clearer identification and evaluation.

## RESULTS

**Figure 1:**
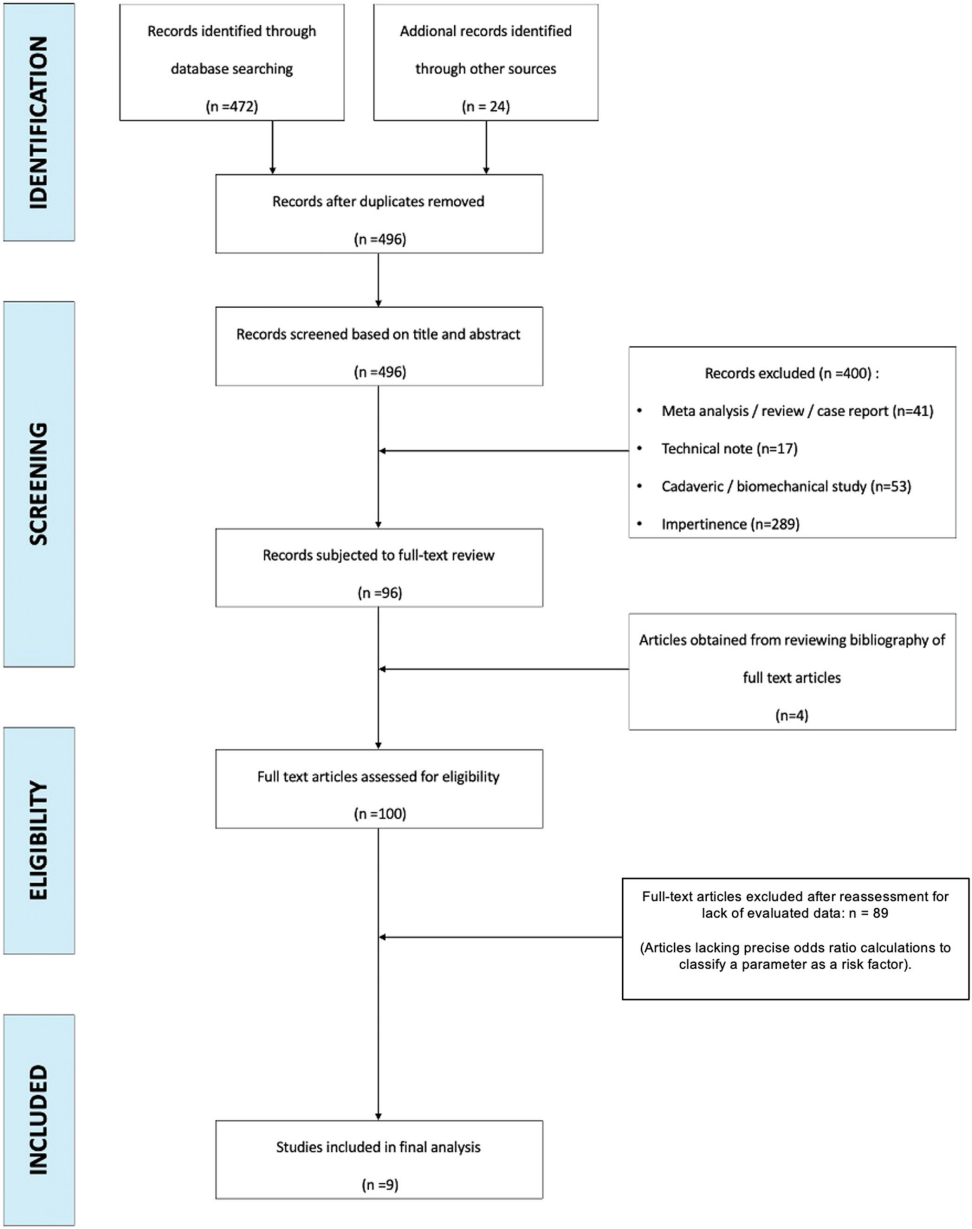
Flowchart of inclusion and analysis.

The initial PubMed/Ovid MEDLINE database search identified 496 articles (Figure 1). No duplicates were found, and all these 496 titles and abstracts were screened. One hundred articles entered the phase of full‐text review, and their references were screened for possible eligible articles. All references identified were obtained and reviewed independently by two reviewers (R.L. and C.C.H.) from July 2023 to October 2023. Table 1 illustrates the general information about the studies that were included. After considering all the potentially eligible references, 9 (1.8%) of the studies met all the inclusion criteria [31, 33, 40, 42, 46, 53, 55, 56, 57] (Table 1). All nine of these studies had a level of evidence at either 3 or 4, and were retrospective case series (Table 1).

**Table 1:** Study details.

	Year of publication	Journal	Country	Level of study	Study type
Li et al. [31]	2009	AJSM	USA	IV	Case series
Petrera et al. [42]	2014	AJSM	Canada	IV	Case series
Park et al. [40]	2016	AJSM	South Korea	III	Cohort study
Xu et al. [53]	2016	AJSM	Korea	III	Cohort study
Yoshimoto et al. [56]	2022	FAI	Japan	III	Retrospective comparative study
Luthfi et al. [33]	2023	FAI	Japan	IV	Retrospective case series
Su et al. [46]	2023	FAI	China	III	Retrospective cohort study
Yoshimoto et al. [55]	2023	KSSTA	Japan	IV	Retrospective case series
Yoshimoto et al. [57]	2023	FAI	Japan	III	Retrospective comparative study

Abbreviations: AJSM, The American Journal of Sports Medicine; FAI, Foot & Ankle International; KSSTA, Knee Surgery Sports Traumatology Arthroscopy.

The total sample size was 762 patients. The sample size of each study ranged from 49 to 199 patients. Surgery was considered in all articles in cases of failed medical treatment, with no discrepancies reported in this regard. Additionally, all procedures were Broström‐Gould repairs without the use of suture tape reinforcement. Eighty‐nine patients (11%) had treatment failure as defined by recurrence of instability ranged from 5.7% to 28.5%. The age of the patients among the different study populations ranged from 13 to 76 years. In this review, all the articles had a minimum of 1‐year follow‐up, while a 5‐year follow‐up was reported in only 1 out of 9 studies (11%) [40] (Table 2).

**Table 2:** Participant characteristics.

References	Ankle, *n*	Age (years)*	Gender ratio (M/F)	Follow‐up (months)*	Approach for surgery	Recurrence rate % (*n*)
Li et al. [31]	52	19.6	NR	29	Open	5.7 (3)
Petrera et al. [42]	49	25 (18–37)	23/26	21 (24–60)	Open	6.1 (3)
Park et al. [40]	199	29	136/63	60 (48–108)	Open	8 (16)
Xu et al. [53]	100	35	56/44	43	Open	6 (6)
Yoshimoto et al. [56]	57	38.9 (13–76)	21/9	16.7 (12–36)	Arthroscopic	17.5 (10)
Luthfi et al. [33]	56	31.8 (14–62)	26/30	15.6 (12–41)	Arthroscopic	28.5 (16)
Su et al. [46]	118	34.6 (24–45)	78/40	30.2 (26–36)	Arthroscopic + Open	7.6 (9)
Yoshimoto et al. [55]	68	39.2 (11–73)	33/34	15.8 (12–36)	Arthroscopic	19.1 (13)
Yoshimoto et al. [57]	63	46 (22–61)	31/32	24 (18–31)	Arthroscopic	20.6 (13)

Abbreviations: NR, not report; SD, standard deviation.

* Data are presented as mean ± s.d. or as mean (range).

The distribution of risk factors found in each study is shown in Table 3 divided into three categories (patient factor, imaging assessment and surgical finding) to allow easier identification and assessment. Generalized joint laxity (GJL), high level of sporting activities and female gender were reported to be patient factors which can lead to recurrence of instability after surgical repair of lateral ankle ligaments. GJL was reported by three studies while high level of sporting activities was reported by two studies. Three articles proposed that varus hindfoot alignment was associated with higher risk for recurrent ankle instability after lateral ankle ligament repair. Varus hindfoot alignment was included in the category of imaging assessment in view of the objective measurements of the hindfoot alignment provided based on weightbearing radiographs in the three articles. Additionally, surgical findings such as poor quality of the remnant lateral ligaments (anterior talofibular ligament; ATFL and calcaneofibular ligament; CFL) leading to recurrence of instability were reported by three articles while syndesmosis widening diagnosed intraoperatively was suggested in one article to contribute to sprain recurrence.

**Table 3:** Synthesis of risk factors by categories for the recurrence of instability after lateral ankle ligament repair found in the literature.

Patient	Generalized joint laxity	Park et al. [40]; Xu et al. [53]
	High level of sporting activities	Luthfi et al. [33]
Imaging assessment	Hindfoot alignment/Varus tilt	Yoshimoto et al. [56]; Yoshimoto et al. [57]
Surgical finding	Remnant ATFL quality	Luthfi et al. [33]; Yoshimoto et al. [55]
	CFL injury	Luthfi et al. [33]
	Intraoperative syndesmosis widening	Su et al. [46]

Abbreviations: ATFL, anterior talofibular ligament; CFL, calcaneofibular ligament.

## DISCUSSION

The main finding of this study was the identification of six risk factors for recurrent instability after lateral ankle ligament repair: generalized joint laxity, high‐level sporting activities, hindfoot varus alignment, poor remnant ATFL quality, CFL injury, and intraoperative syndesmosis widening. They were organized into three categories to facilitate understanding and patient assessment (Table 3). These categories are intended to guide management decisions for treating surgeons and physicians. To our knowledge, this is the first systematic review to provide an updated summary of all the risk factors for recurrent instability following lateral ankle ligament repair.

The most commonly cited risk factor for treatment failure was GJL [40, 42, 53]. Park et al. [40] reported a recurrence rate of 23.8% in patients with GJL versus 3.8% in those without GJL, along with poorer Karlsson scores after the modified Broström procedure. Similarly, Li et al. [31] found recurrence rates of 11.4% in patients with GJL compared to 1.8% in those without, in a cohort matched for age and BMI. High‐level sporting activities and female gender were also notable risk factors. Luthfi et al. [33] showed that patients with higher preoperative ankle activity scores (AAS) were more likely to experience instability recurrence, while lower AAS was associated with no recurrence. Thes et al. [48] identified female gender as an independent risk factor for instability‐related failure in a cohort of 172 patients, though this study included both ligament repair and reconstruction cases. However, other studies [17, 35] found no significant gender differences in failure rates after primary ligament repair, although they defined failure as poor outcome scores rather than instability recurrence.

These non‐modifiable patient risk factors can be managed in two ways. First, patient expectations should be addressed preoperatively, and the potential for recurrence of instability must be explained during the consent process. Second, surgical approaches can be adjusted by augmenting the repair with a suture‐tape construct to improve the stability and durability of the lateral ankle ligament repair [9, 23, 29, 40]. Cho et al. [9] demonstrated this in a study of 28 patients with generalized ligamentous laxity, where a modified Broström procedure augmented with suture‐tape led to significant improvements in FAOS and FAAM scores, with minimal recurrence of instability at 2‐year follow‐up. Alternatively, tendon graft reconstruction may be considered to further enhance stability and reduce the risk of repair failure [23, 29, 40, 54].

Hindfoot varus alignment is a noted risk factor for failure of lateral ankle ligament repair [16, 45]. Yoshimoto et al. [55] found that 13 out of 63 ankles with recurrent instability after arthroscopic lateral ligament repair for CLAI had high preoperative tibiocalcaneal angles, indicating varus alignment [30]. Those with a tibiocalcaneal angle ≥ 2.7° had significantly higher recurrence rates and lower pain subscale scores on the SAFE‐Q. Similarly, Luthfi et al. [33] reported that 16 out of 56 patients with higher preoperative and postoperative talar tilting angles experienced recurrent instability. A varus‐tilted tibial plafond was also an independent predictor of recurrent instability and lower pain scores in 10 out of 57 patients [56]. Correcting hindfoot varus malalignment may help prevent recurrence, although in professional athletes, the risk of prolonged downtime and altered performance should be carefully considered [54].

The quality of the remnant ATFL is a risk factor for poor outcomes and treatment failure [14, 55]. It can be assessed preoperatively via imaging or intraoperatively [41]. Park et al. [41] found that outcomes after the modified Broström procedure were similar, regardless of ATFL presence on MRI, ultrasound, or arthroscopy, with good FAOS scores at an average follow‐up of 30.1 months. Feng et al. [14] corroborated this, showing no significant difference in postoperative functional outcomes between 49 patients with ATFL remnant repair and 35 without. In contrast, Yoshimoto et al. [55] found that poor‐quality ATFL remnants (high T2 signal) on MRI or arthroscopy led to recurrence of instability despite similar SAFE‐Q scores. They noted a difference in defining treatment failure, with others using poor functional scores, while they considered recurrence of instability as failure [55]. To mitigate this risk, augmentation with suture‐tape or primary reconstruction with tendon graft may be considered [23, 29, 40, 54].

Severe syndesmosis widening can negatively affect outcomes after the Broström procedure for CLAI [46]. Su et al. [46] found that CLAI patients with middle tibiofibular syndesmosis widening of ≥ 4 mm had delayed return to work and sports, lower rates of returning to preinjury sports, and recurrence of sprains. The instability of the syndesmosis joint can lead to fibula displacement, rotatory instability, and increased diastasis and talar tilt, resulting in re‐sprains [46]. Therefore, intraoperative examination of the syndesmosis is crucial, as untreated syndesmosis instability is a risk factor for treatment failure. We agree with the authors that syndesmosis instability should prompt consideration of simultaneous stabilization to prevent recurrence of instability.

This systematic review highlights the dilemma of defining treatment failure in studies on risk factors for recurrence of instability after lateral ligament repair. Recurrence is sometimes considered a distinct entity [3, 33], while other times it's viewed as a complication [9] or failure [48]. Some authors differentiate between isolated lateral ankle sprain recurrence and CLAI recurrence [9, 10], while others report instability that improves or disappears [47]. Dallman et al. [12] noted this confusion and conducted a systematic review on treatment failure definitions. They found failure rates for the Broström‐Gould technique ranged from 1.1% to 45.2%, influenced by differing definitions. They proposed a uniform definition of failure, including specific physical examination findings, dynamic stability assessments, and FAAM evaluations to reduce ambiguity.

This study has several limitations, particularly in analyzing risk factors for recurrence of instability after lateral ankle ligament repair. A key limitation is the lack of a formal Risk of Bias Assessment for the included studies, which could have strengthened the reliability of our findings. Additionally, confusion between “repair” and “reconstruction” required a thorough review of clinical series [1, 7, 28, 37, 43], resulting in 100 eligible articles (Table 1). Recurrence rates are inconsistently reported, with some studies lacking specific data. Recurrence after lateral ligament repair is infrequently noted in small case series, complicating statistical analysis. Some known factors, such as symptom duration or subfibulare ossicles larger than 1 cm, were not reported [27]. The overall evidence level (all Level 3 and 4 studies) raises concerns about potential methodological bias. Furthermore, data heterogeneity limited meaningful analysis of the impact of Gould modification on recurrence rates, and only two studies reported follow‐up durations over five years, a period during which repair outcomes often deteriorate [44, 50]. Despite these limitations, this remains the first comprehensive review of risk factors for recurrence after lateral ankle ligament repair for CLAI, providing evidence‐based recommendations for improving patient outcomes.

## CONCLUSION

The presence of risk factors such as GJL, high‐level sports activities, female sex, varus hindfoot alignment, poor ligament quality, and intraoperative syndesmosis widening should guide surgical strategy to reduce the risk of treatment failure in lateral ankle ligament repair for CLAI.

## AUTHOR CONTRIBUTIONS

**Ronny Lopes**: Article design; data collection and analysis; article writing. **Choon Chiet Hong**: Article design; data collection and analysis; article writing. **James Calder**: Article writing; reviewing; and editing. **Gino M. M. J. Kerkhoffs**: Article writing; reviewing; and editing.

## CONFLICT OF INTEREST STATEMENT

Ronny Lopes does consulting for Arthrex/Serf Extremity unrelated to this study, Choon Chiet Hong received remuneration for speaking in educational program/lectures by Arthrex unrelated to this study, James Calder received remuneration for speaking in educational program/lectures by Arthrex unrelated to this study, and Gino M. M. J. Kerkhoffs does consulting for Arthrex unrelated to this study.

## ETHICS STATEMENT

Ethics approval and consent to participate were not required for this study, as it is a systematic review of previously published studies. The review adhered to PRISMA (Preferred Reporting Items for Systematic Reviews and Meta‐Analyses) guidelines.

## Data Availability

All data generated or analyzed during this systematic review are included in this published article and its supplementary information files. No primary data collection was conducted, as the study is based on previously published literature.
